# Semi-automated analysis of cerebral capillary red blood cell velocities allows modeling of transit time distribution after experimental subarachnoid hemorrhage in mice

**DOI:** 10.1117/1.NPh.12.S1.S14612

**Published:** 2025-05-15

**Authors:** Kévin Chalard, Yan Chastagnier, Julie Perroy, Vivien Szabo

**Affiliations:** a University of Montpellier, IGF, CNRS, INSERM, Montpellier, France; b CHU Montpellier, Department of Critical Care and Anesthesiology Gui de Chauliac, Montpellier, France; c University of Montpellier, LIRMM, CNRS, Montpellier, France; d University of Montpellier, L2C, CNRS, Montpellier, France

**Keywords:** optics, subarachnoid hemorrhage, cerebral blood flow

## Abstract

**Significance:**

Microvascular dysfunction stems from the origin of various neurological diseases. Among these, delayed cerebral ischemia following subarachnoid hemorrhage (SAH) is a major complication. Even though pathogenesis remains poorly understood, hypotheses converge toward early and persistent microvascular dysfunction. In this context, mathematical models have been developed to study oxygen delivery using theoretical distributions of capillary flux. However, these distributions lack experimental validation.

**Aim:**

We propose experimental recording of capillary red blood cell (RBC) velocities in a superficial cortical microvascular network in a mouse model of SAH, testing theoretical transit time distributions and their implications on tissue oxygenation.

**Approach:**

We performed optical recording of RBC velocities. We propose a complete software, available on GitHub, for velocity semi-automated measurement. Experimental data were fitted with Gamma and Cauchy probability distribution functions (PDFs). Corresponding maximal oxygen metabolic rates (${\mathrm{CMRO}}_{2}^{\max}$) were computed.

**Results:**

Data showed that transit time distributions changed after SAH, such that they followed a Cauchy distribution. Corresponding ${\mathrm{CMRO}}_{2}^{\max}$ maps showed a malignant capillary heterogeneity state.

**Conclusions:**

We provide distributions of transit times in an SAH mouse model, allowing us to discuss PDF implications for maximal oxygen consumption.

## 1 Introduction

### 1.1 Microvascular Dysfunction in Neurological Disease

To function, the brain needs constant oxygen and metabolic supply. Because of such a requirement, it has evolved appropriate vascular macro and microarchitecture together with regulatory mechanisms.^1^^–^^6^ When these components pathologically dysfunction, supply impairment ultimately leads to a variety of brain diseases, ranging from acute no-reflow after ischemic stroke to chronic neurodegeneration in Alzheimer’s disease.^4^^,^^5^^,^^7^^,^^8^

Within the nosographical spectrum of neurological ischemic affections, delayed cerebral ischemia (DCI) after aneurysmal subarachnoid hemorrhage (SAH) is a singular entity. Indeed, it constitutes a dramatic complication that occurs several days after the initial stroke,^9^^,^^10^ such that it would seem preventable. Unfortunately, pathophysiological understanding of this secondary infarction is nebulous,^10^ such that only empirical treatments are available.^10^^–^^12^

Among the multiple hypotheses postulated to explain DCI, early and persistent microvascular dysfunction would be supported by both theoretical and experimental evidence.^13^^–^^24^

### 1.2 Modeling of Oxygen Supply

In the context of SAH/DCI, modeling of oxygen and metabolic brain supply is of fundamental interest because it allows theoretical study of pathogenesis, and as such, has received increasing attention.^17^^,^^25^^,^^26^ In any of these models, oxygen extraction into brain tissue depends on red blood cell (RBC) flux distribution in the capillary network, which emerges from the underlying vascular microarchitecture.^25^^,^^27^^–^^29^ Several models of RBC transit time distribution are currently being used in the literature, for instance, Gamma,^27^^,^^28^ Cauchy,^29^ and a number of other distributions,^28^ having distinctive consequences on the calculated maximal cerebral oxygen metabolic rate (${\mathrm{CMRO}}_{2}$).^28^ Still, no conclusive experimental evidence allows to decide which may be more suitable, further justifying that models of transit time distribution need recording of capillary flux for validation.^25^^,^^28^

### 1.3 Recording of Capillary Flux to Model Transit Time Distribution

Several optical methods have been developed and successively used to record RBC velocities in cerebral capillaries. An exhaustive review of these methods is beyond the scope of the present work such that only a brief overview is presented hereafter. Notably, widefield microscopy was used in the 1960s by Rosemblum to first describe pulsatility changes in RBC velocities corresponding to the cardiac cycle from tracking erythrocytes near the arterioles walls in pial microcirculation.^30^ Line-scanning confocal fluorescence microscopy later enabled deeper imaging of a limited number of sampled capillaries at high acquisition rates, allowing for the measurement of the effect of ${\mathrm{CO}}_{2}$-inhalation on brain geometry and capillary blood flow dynamics in rats.^31^ This technique also demonstrated that the characteristics of RBC velocities in mice were similar to those in rats, in terms of absolute values and distributions.^32^ Nowadays, two-photon laser scanning microscopy is a widely used method due to its high imaging depth, temporal and spatial resolution, and excellent signal-to-noise ratio.^33^^–^^35^ However, the number of sampled capillaries remains limited to a few tenths per animal. In experimentally induced SAH in mice, it has been reported that 40 capillaries were sampled from a total of four animals in a pioneering study^20^ and 150 capillaries from a total of eight animals in a subsequent one.^21^

To model RBC transit time distribution in the capillary network, it is necessary to record from a larger number (i.e., several hundreds) of vessels in single animals because the distribution probability density function (PDF) could be affected by the dispersion arising from pulling data across multiple animals, or even multiple time points in a single animal. Consequently, automated data processing and analysis are required.

### 1.4 Summary

In the present work, we used a mouse model of SAH.^36^ We recorded RBC velocities from hundreds of superficial cortical microvessels and developed a complete software for semi-automated analysis. This allowed quantitative description of RBC velocities and transit times in single animals, before and after SAH, as well as fitting data with mathematical models. Finally, we conclude on the most likely model and discuss the implications regarding the underlying network and maximal ${\mathrm{CMRO}}_{2}$.

## 2 Material and Methods

All methods are reported in accordance with ARRIVE guidelines.

### 2.1 Animal Care

All animal procedures were conducted in accordance with the European Communities Council Directive. The experimental protocol was approved by the Languedoc-Roussillon Ethical Committee for Animal Experimentation (national registration number: APAFIS 29441-2021012116337609 v2). C57Bl/6J mice were obtained from Janvier Labs and maintained in a 12-h light/dark cycle in stable conditions of temperature (22°C±2°C) and humidity (60%) with food and water provided *ad libitum*. Weight and facial expressions were carefully monitored using a mouse grimace scale (MGS) throughout the experiments.^37^ Efforts were made to minimize the number of animals used and their suffering. Subcutaneous administration of meloxicam (2  mg/mL, 5  mg/kg) was performed if MGS was superior to 1. The animal was to be sacrificed if the MGS remained above six despite analgesic treatment for 24 h, or in the event of prostration or weight loss over 20% for more than 3 consecutive days despite the addition of palatable food gel.

### 2.2 Cranial Window Implantation

The surgical procedure for cranial window implantation was performed under sterile conditions. Anesthesia was induced with isoflurane (4% for induction, 1.5% to 2% for maintenance) saturated with oxygen, and supplemented with intraperitoneal injection of ketamine (10  mg/mL, 60  mg/kg). Analgesia was provided with a subcutaneous injection of meloxicam (2  mg/mL, 10  mg/kg) to minimize post-operative pain. Body temperature was maintained at 37.5°C using a heating pad to prevent hypothermia.

Once the mouse was fully anesthetized, it was positioned in a stereotaxic frame to immobilize the head and allow precise surgical manipulation. The eyes were protected with ophthalmic cream and covered with opaque paper to prevent damage from the operating lights. The scalp was shaved, and the skin was disinfected with an antiseptic solution to reduce the risk of infection. A local anesthetic, ropivacaine (2  mg/mL, 50  μL), was injected subcutaneously at the surgical site to provide additional pain relief during the procedure.

A midline incision was made along the scalp, and retractors were used to expose the parietal bone. The periosteum covering the bone was carefully removed. A craniotomy, 3 mm in diameter, was then performed on the right parietal bone, ensuring that the dura mater remained intact. Once the craniotomy was completed, a cranial window made of a microscope coverslip was placed over the exposed area and cemented using photopolymerizable material (Tetric Evoflow, IVOCLAR VIVADENT, Schaan, Liechtenstein). The skin around the window was sealed with the same dental cement to ensure a secure fit.

After the procedure, the animals were allowed to recover on a heating pad before being returned to their home cages. Mice were closely monitored post-operatively to ensure proper healing and minimize any discomfort or complications.

### 2.3 *In Vivo* Recording from Brain Superficial Microvessels

Optical recordings were performed two weeks after cranial window placement, under general anesthesia. Anesthesia was induced with intraperitoneal injections of xylazine (0.5  mg/mL, 5  mg/kg) and ketamine (10  mg/mL, 100  mg/kg) and maintained with repeated administration of ketamine (30 to 50  mg/kg) every 45 min. Recordings were conducted before and after SAH induction, following a 150-μL retro-orbital intravenous infusion of tetramethylrhodamine isothiocyanate–Dextran of 155-kDa molecular weight (Sigma-Aldrich T1287, St. Louis, Missouri, United States).^38^

An upright fluorescence microscope, the Andor Revolution DSD2 equipped with a 10× water-dipping Olympus objective (numerical aperture 0.3, working distance 3.5 mm), an Andor Metal Halide light source AMH-200-F6S, with a 556/20  nm excitation filter and a 609/54  nm emission filter, and an Andor Zyla 5.5 USB3 sCMOS Camera, was used. This system yields a lateral resolution of 1  μm (see Sec. 1.1 in the Supplementary Material for details). Anatomical acquisitions were performed in differential confocal fluorescence mode. We acquired a series of two-dimensional images taken at different depths along the z-axis, allowing for a three-dimensional reconstruction of the imaged area. The Z-stack covered a volume of ∼2500  μm×2500  μm×150  μm, created by assembling a 3 by 2 mosaic. This mosaic consisted of 6 individual image stacks, each measuring 730  μm×1231  μm×150  μm, acquired at a frame rate of 2 Hz. It was used to select from a three-dimensional volume the fields of view from which time series were to be acquired. Up to four fields of view were selected in each mouse, focusing on regions containing microvessels. A series of images taken over time at a single focal plane (T-stacks) were acquired in widefield mode to maximize the number of capillaries observed. Each T-stack was performed in an acquisition field of 730  μm×570  μm, with an acquisition rate of 100 Hz, lasted for 2 s and was repeated every 5 min in all selected fields of view.

### 2.4 Subarachnoid Hemorrhage Induction Procedure

We followed the protocol described by Sabri and colleagues to induce SAH.^36^ The procedure was conducted under general anesthesia, using the same surgical steps as outlined in the cranial window implantation section. A craniotomy of ∼1  mm in diameter was drilled 4.50 mm anterior to Bregma and slightly off midline (+0.30  mm) to avoid the sagittal sinus. Blood was drawn from a litter-mate to be used for the injection. A 30G needle was then inserted at a 40-deg angle ventrally through the burr hole, advancing 6 to 9 mm until contact was made with the skull base. The needle was then withdrawn by 0.5 mm to position the tip in the prechiasmatic cistern. Finally, a 75-μL injection of blood was administered through the intracisternal needle over 7.5 s, with a controlled flow rate of 10  μL/s.

### 2.5 Data Processing and RBC Velocity Measurement

To correct motions of the field of view during acquisition, caused by respiratory cycles, and between acquisitions, we performed a rigid registration inside each T-stack and between the T-stacks.

**Fig. 1:**
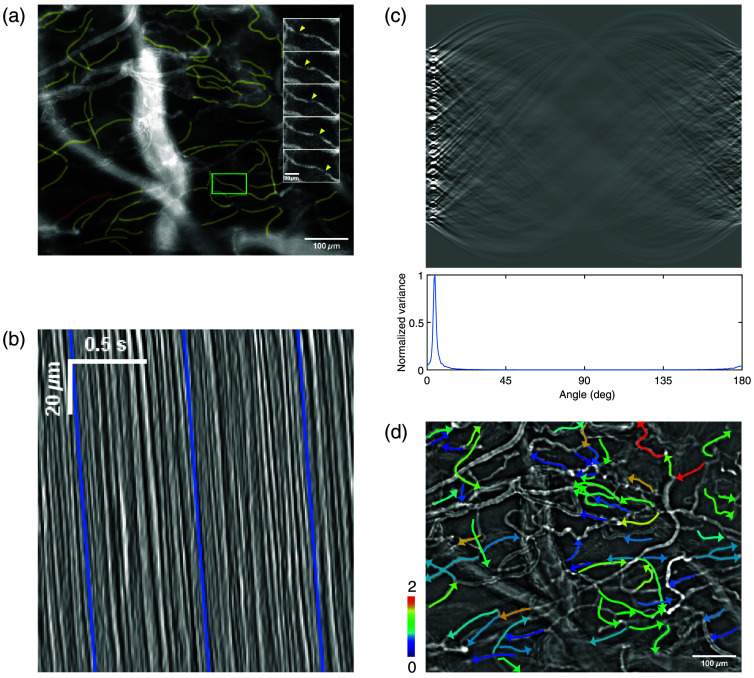
Work-flow for RBC velocity measurement. (a) Raw image with manually drawn ROIs. The inset corresponds to five consecutive time points of the green rectangle. Arrowheads point to a red blood cell in motion. (b) Profile-time image of the red ROI from panel (a), after a horizontal Sobel filtering. Blue lines indicate the angle found by the following process, allowing manual verification. (c) Top, radon transform of the profile-time image from panel (b). Bottom, plot of the normalized variance of each column from radon transformed image. The peak variance gives the angle of the streaks. (d) Contrast-enhanced image of the stack with ROIs drawn as arrows to indicate direction, with color coding of the velocity, in mm/s.

We manually drew regions of interest (ROIs) on one of the stacks [Fig. 1(a)]. ROIs consisted of poly-lines or freehand lines that followed a microvessel with a width of 5  μm. The same ROIs were used for all stacks corresponding to the same field of view. The ROI position was manually adjusted from stack to stack when necessary.

In every field of view, vessels were categorized as microvessels when RBCs flew into them one by one, in a single file. To maximize the probability that the microvessels were capillaries, we kept for analysis those who emerged from divergent input vessels, and converged into output vessels.

For each ROI and each stack, a profile-time image was generated [Fig. 1(b)]. The profile of the linear ROI was averaged over its width and mapped to a column of the profile-time image, each column of the image corresponding to a different time frame of the original stack.

To remove horizontal stripes due to inhomogeneous illumination along the vessel, the image was preprocessed. For each horizontal line, the average and standard deviation of the pixels’ intensity were computed. For each pixel, the average intensity of the corresponding line was subtracted, and the result was divided by the standard deviation of the line intensity.

On profile-time images, RBCs leave dark streaks [Fig. 1(b)]. Those streaks represent the displacement of an RBC during a certain amount of time, from which can be inferred the velocity. We measured the angle of those streaks, for which the tangent is the ratio between time and distance, and deduced the velocity of the RBC. To do so, Radon transform^39^^,^^40^ was applied to the preprocessed image for each angle between 0 and 180 deg by steps of 1 deg. The variance of the resulting projections was computed, and its maximum was determined [Fig. 1(c)]. To refine the position of the maximum, a second-order polynomial fit was performed using the maximum and its two neighbors. The peak of that parabola gave the angle $\theta_{1}$.

The same operation was done again for each angle between $\theta_{1}-1$ and $\theta_{1}+1$ by steps of 0.01 deg, giving $\theta_{f}$.

The velocity was determined using the equation: $v=\mathrm{cotan}(\theta_{f})*\frac{{\mathrm{res}}_{y}}{{\mathrm{res}}_{x}}$, where ${\mathrm{res}}_{y}$ is the pixel resolution of the original stack and ${\mathrm{res}}_{x}$ is its temporal resolution.

We then manually verified the measured angles by automatically drawing a line at that angle and visually comparing it with the streaks. (1) Angles correctly measured were kept untouched. (2) For angles where the line and streaks were not parallel, the line was manually redrawn. The angle and velocity were updated according to the new line orientation. (3) For angles where no streaks were visible, stacks were opened for further verification. A horizontal line was drawn when verification revealed an interruption of flow. In this case, the angle and velocity were updated. Otherwise, the values were discarded.

For a velocity to be considered measurable, RBC must be visible in the ROI on at least two frames. In practice, the corresponding minimum angle is defined as $\theta_{\min}=\mathrm{atan}(\frac{2}{\text{length}(\mathrm{ROI})})$ and $|\theta |>\theta_{\min}$. We discarded values for which that criterion was not achieved.

Because RBC displacement is measured in the imaging plane only, the method underestimates RBC velocities in vessels tilted relative to the imaging plane. To quantify this, we measured the tilt angle (see Sec. 1.2 in the Supplementary Material).

In the sCMOS camera, each line of the sensor is typically not exposed at the exact same time, as the rolling shutter of the camera is moving from line to line. It follows that any ROI that begins and ends at different positions of the rolling shutter needs a slight adjustment in the velocity value. To compensate for this effect, we multiplied the velocity by $\frac{t}{t+\epsilon_{t}}$, where t is the time for a RBC to go through the ROI and $\epsilon_{t}$ is the time for the rolling shutter to go from the beginning of the ROI to its end. $\epsilon_{t}$ is positive if the ROI follows the rolling shutter and negative if they go in opposite directions.

From here on, for each mouse and at each time point, data coming from the different fields of view were pooled.

### 2.6 Statistical Analysis

Because distributions of transit times and velocities were not normal, a Kruskal–Wallis test was used to compare distributions at the different time points.

### 2.7 Data Fitting with Mathematical Models

Transit times were computed as capillary length divided by velocity, using three different assumptions. The first one was used in the subsequent computations, whereas the following two are presented as the Supplementary Material. (1) The ROI length was used as capillary length. (2) The capillary length is constant for all capillaries and has a value of 50  μm, as in the work from Goirand et al.^25^ (3) Capillaries were randomly assigned a length from a log-normal distribution with a median of 50  μm ranging roughly from 10 to 200  μm (μ=ln(50), σ=ln(1.6)), as reported by Blinder et al.^41^

Three periods of time were considered. We chose the first data point for each mouse as the baseline. Two other time points were selected at around 45 and 85 min post-SAH. For each of the time points and each mouse, velocity distributions were fitted using inverse Gamma^17^ and Cauchy^25^ PDFs, whereas transit time distributions were fitted using Gamma, Inverse Gamma, Inverse Gaussian, Log-normal,^28^ and Cauchy PDFs.^25^

Fit procedure minimized the modified sum of squares defined in the following equation:

$$\mathrm{MSS}=\underset{i}{\sum }\frac{(y_{i}-f_{i}{)}^{2}}{f_{i}},$$

where $f_{i}$ is the fit function, $y_{i}$ is the data point, and i is the set of bin values. For velocities distribution fit, bins ranged from 0 to 3000 with a size of 50  μm/s. For transit time distribution fit, bins ranged from $10^{-1.7}$ to $10^{2}$, $10^{-1.5}$ to $10^{1.6}$, or $10^{-2.2}$ to $10^{2}\;s$, respectively, for ROI length, constant 50  μm or random capillary length distributions, with a logarithmic spacing of 10 bins per decade.

The $R^{2}$ displayed on transit times fit figures was computed as

$$R^{2}=1-\frac{{\mathrm{MSS}}_{\mathrm{res}}}{{\mathrm{MSS}}_{\mathrm{tot}}},$$

where ${\mathrm{MSS}}_{\mathrm{res}}$ and ${\mathrm{MSS}}_{\mathrm{tot}}$ are the modified sum of squares for the fit function and data average function, respectively.

To fit the Cauchy function, as velocities were taken as absolute values, the Cauchy function was renormalized so that its area under the curve was equal to 1 on $R^{+}$.

The following PDFs were used in the analysis:

– Gamma PDF
  $$f(x;\alpha ,\beta )=\frac{1}{\Gamma (\alpha )\beta^{\alpha }}x^{\alpha -1}e^{-x/\beta },$$
  where α is the shape parameter and β is the scale parameter.
– Inverse Gamma PDF
  $$f(x;\alpha ,\beta )=\frac{\beta^{\alpha }}{\Gamma (\alpha )}x^{-\alpha -1}e^{-\beta /x},$$
  where α is the shape parameter and β is the scale parameter.
– Cauchy PDF
  $$f(x;\mu ,\gamma )=\frac{1}{\pi \gamma [1+(\frac{x-\mu }{\gamma }{)}^{2}]},$$
  where μ is the location parameter and γ is the scale parameter.
– Inverse Gaussian PDF
  $$f(x;\mu ,\lambda )=\sqrt{\frac{\lambda }{2\pi x^{3}}}e^{-\frac{\lambda (x-\mu {)}^{2}}{2\mu^{2}x}},$$
  where μ is the mean and λ is the shape parameter.
– Log-normal PDF
  $$f(x;\mu ,\sigma )=\frac{1}{x\sigma \sqrt{2\pi }}e^{-\frac{(\log(x)-\mu {)}^{2}}{2\sigma^{2}}},$$
  where μ is the logarithm of location and σ is the logarithm of scale.

### 2.8 ${\mathrm{CMRO}}_{2}^{\max}$ Calculation

${\mathrm{CMRO}}_{2}^{\max}$ was computed following the method described by Jespersen and Østergaard:^27^

$${\mathrm{CMRO}}_{2}^{\max}=\mathrm{CBF}*\mathrm{Ca}*{\mathrm{OEF}}^{\max},$$

where CBF is the cerebral blood flow, Ca represents the arterial oxygen concentration, and ${\mathrm{OEF}}^{\max}$ is the maximal oxygen extraction fraction.

CBF is calculated as

$$\mathrm{CBF}=\frac{\mathrm{CB}V^{\prime }}{\mathrm{MTT}},$$

with $\mathrm{CB}V^{\prime }$ being the capillary blood volume and MTT the mean transit time.

To compute ${\mathrm{OEF}}^{\max}$, the following integral was used:

$$OEF^{\max}=\int_{0}^{\infty }h(\tau )Q(\tau )d\tau$$

where h(τ) represents the transit time distribution and Q(τ) is the oxygen extraction for a single capillary. The oxygen extraction function Q(τ) is defined as^27^

$$Q(\tau )=1-e^{-k\tau },$$

with k the forward and reverse rate constant of oxygen.

Using the same assumptions as Jespersen and Østergaard,^27^ we set Ca to 19  mL/100  mL, and $\mathrm{CB}V^{\prime }$ to 1.6%. The value of k was set to $1.5\;s^{-1}$ as in the work from Goirand et al.^25^

Numerical integration was performed to compute ${\mathrm{OEF}}^{\max}$. For experimental data, the equation was simplified to

$${\mathrm{OEF}}^{\max}=\frac{1}{N}\underset{\tau }{\sum }Q(\tau ),$$

where N is the number of vessels. For modeled data, the equation becomes

$${\mathrm{OEF}}^{\max}=\underset{\tau }{\sum }h(\tau )Q(\tau ).$$

Here, τ was discretized into intervals of 0.001 s, ranging between 0 and 30 s.

Finally, ${\mathrm{CMRO}}_{2}^{\max}$ was computed as

$${\mathrm{CMRO}}_{2}^{\max}=\frac{\mathrm{CB}V^{\prime }}{\mathrm{MTT}}*\mathrm{Ca}*{\mathrm{OEF}}^{\max}=\frac{0.016}{\mathrm{MTT}}*19*{\mathrm{OEF}}^{\max}.$$

In the case of the Cauchy distribution, as the mean is undefined, MTT was set to the mean over the interval of interest (0 to 30 s).

We computed ${\mathrm{CMRO}}_{2}^{\max}$ maps for the Gamma and Cauchy models by varying the mean and standard deviation of the Gamma PDF and the location and scale parameters of the Cauchy PDF. When using the Cauchy PDF, the upper limit of the integration range impacts ${\mathrm{CMRO}}_{2}^{\max}$ values. However, it does not change the general shape of the map nor the existence of a malignant heterogeneity state.

## 3 Results

We recorded brain capillaries from anesthetized mice using an upright microscope, before and after SAH induction. This allowed collecting data from 68, 142, and 223 capillaries from mouse m1, m2, and m3 before SAH, to 48, 158, and 222 capillaries at T≈45  min and 46, 116, and 221 capillaries at T≈85  min from each mouse after SAH, respectively. Data were analyzed following the procedure described in Sec. 2.5 and illustrated in Fig. 1. Briefly, linear ROIs were manually drawn along the vessels. Only ROIs traced over presumed capillaries were analyzed. Profile-time images were generated, on which RBC temporal trajectory in the capillary appeared as dark streaks [see Sec. 2.5, the Radon transform of this image was computed to find the angle θ of the streaks; velocity v was computed based on this angle (v∝cotan(θ)].

**Fig. 2:**
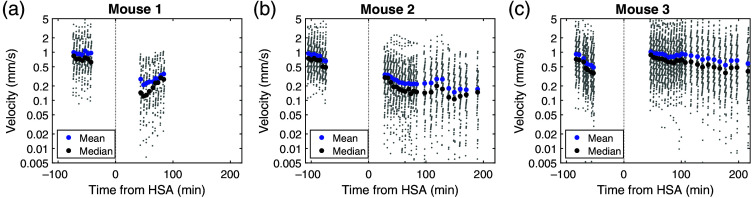
RBC velocities in superficial cortical networks. (a)–(c) RBC velocities against time, before and after subarachnoid hemorrhage in each animal, displayed on a semi-logarithmic scale.

The procedure allowed us to measure RBC velocities in every capillary at every time point, as presented in Figs. 2 and Fig. S2 in the Supplementary Material. Medians of uncertainty due to spatiotemporal resolution and underestimation resulting from tilted vessels were 1.22% and 0.6%, respectively (Fig. S1 in the Supplementary Material). Mean and median maximal measurable RBC velocities, which depend on the spatiotemporal resolution and ROI length (see Sec. 2.5), were 4.76 and 4.28  mm/s, respectively.

Recordings show that velocity distributions changed after SAH (mouse m1, $p=1.02*10^{-14}$, m2, $p=3.04*10^{-42}$, m3, $p=6.67*10^{-5}$, Kruskal–Wallis test, see Sec. 2.6). Mean velocity went from 0.99, 0.94, 0.91  mm/s at T≈−90  min to 0.28, 0.27, 1.02  mm/s at T≈45  min and 0.35, 0.22, 0.81  mm/s at T≈85  min.

**Fig. 3:**
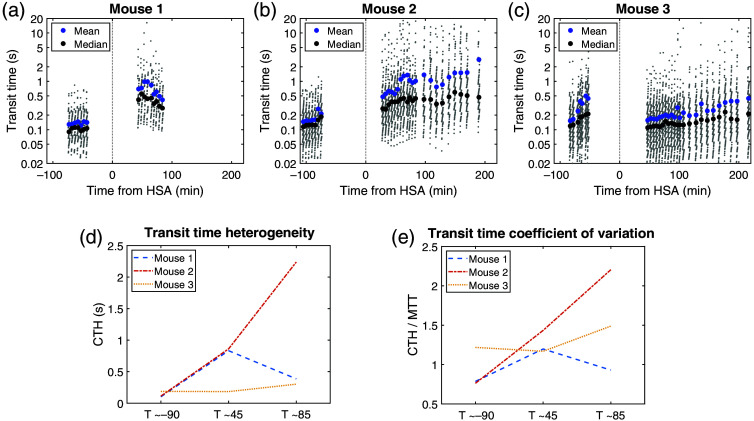
Characterization of RBC transit times over time, before and after subarachnoid hemorrhage. (a)–(c) RBC transit times against time in each animal, displayed on a semi-logarithmic scale. (d) Capillary transit time heterogeneity. (e) Transit time coefficient of variation.

Theoretical models calculating maximal ${\mathrm{CMRO}}_{2}$ make use of capillary RBC transit time distribution.^17^^,^^25^ Hence, following the method described in Sec. 2.7, we inferred transit times from velocities in the three animals [Figs. 3(a)–3(c), Figs. S4 and S5 in the Supplementary Material]. Besides, transit time dispersion has been hypothesized to hinder maximal ${\mathrm{CMRO}}_{2}$.^17^ We quantified this dispersion by computing the capillary transit time heterogeneity (CTH) as the standard deviation of data, including the transit time coefficient of variation (CTH/MTT).

Transit time distribution changed after SAH in the three animals (mouse m1, $p=1.29*10^{-15}$, m2, $p=1.89*10^{-32}$, m3, $p=1.30*10^{-3}$). MTT went from 0.13, 0.15, and 0.15 s at T≈−90  min to 0.69, 0.60, and 0.16 s at T≈45  min and 0.41, 1.02, and 0.20 s at T≈85  min, respectively. CTH/MTT went from 0.79, 0.76, and 1.2 at T≈−90  min to 1.2, 1.4, and 1.2 at T≈45  min and 0.93, 2.2, and 1.5 at T≈85  min, respectively [Fig. 3(c)].

**Fig. 4:**
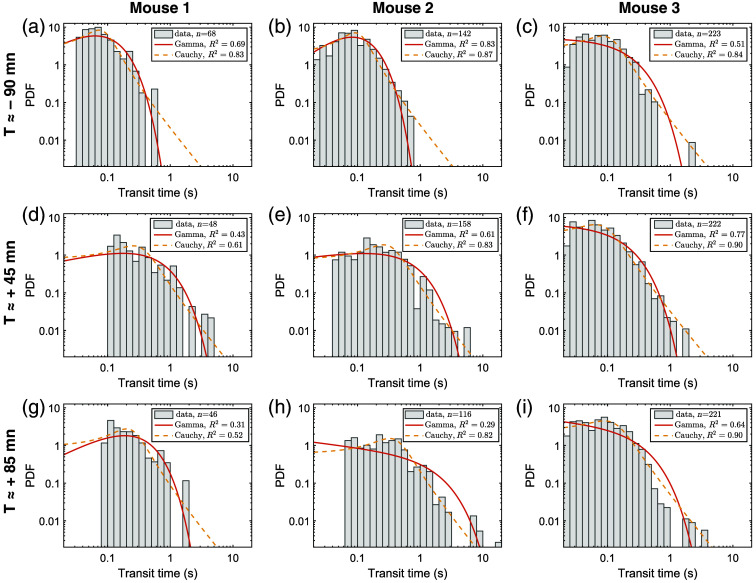
RBC transit time distribution fitted with Gamma and Cauchy PDFs. (a)–(c) First time point for each mouse at baseline, before SAH induction. (d)–(f) Second timepoint selected at around 45 min after SAH induction. (g)–(i) Third timepoint selected at around 85 min after SAH induction.

Finally, transit times PDF allows inference about the underlying microvascular network structure and maximal ${\mathrm{CMRO}}_{2}$ calculation.^27^^–^^29^ Therefore, we fitted putative PDFs^25^^,^^27^ to the obtained data (Fig. 4 and Fig. S6 in the Supplementary Material, see Fig. S3 in the Supplementary Material for fits of the velocity distributions). The results showed that although a Gamma PDF^27^ provided satisfactory results before SAH, it failed to take into account the longer transit times arising after SAH. A Cauchy PDF^29^ fitted more appropriately with both pre- and post-SAH data.

**Fig. 5:**
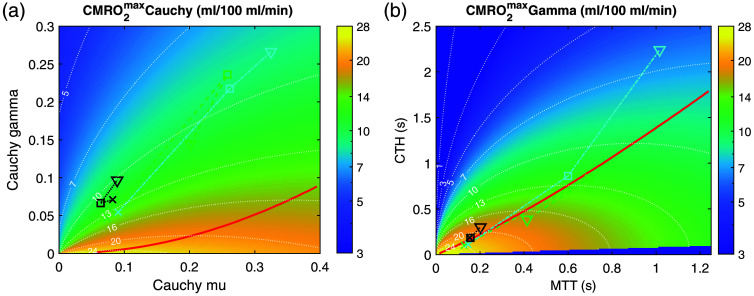
Maximal ${\mathrm{CMRO}}_{2}$ maps. Maximal ${\mathrm{CMRO}}_{2}$ maps for Gamma (a) and Cauchy (b) distributions of capillary transit times. Red lines show the malignant heterogeneity threshold. Color lines show maximal ${\mathrm{CMRO}}_{2}$ calculated from experimental data in mouse 1 (green dashed line), 2 (blue dash-dotted line), and 3 (black dotted line) at first time point (cross symbol), and around 45 (square) and 85 (triangle) min after SAH.

Using conservative assumptions,^27^ we computed ${\mathrm{CMRO}}_{2}^{\max}$ maps for Gamma and Cauchy PDFs, as a function of MTT and CTH/MTT, or location and scale parameters, respectively (Fig. 5). Both these maps show a “malignant CTH” threshold, materialized on the figure as a red line.

## 4 Discussion

### 4.1 Summary

In this study, we used a well-described and widely used mouse model for experimental SAH.^36^^,^^42^^,^^43^ Optical imaging with a widefield epifluorescence microscope allowed for *in vivo* recording from unprecedented numbers of superficial cortical microvessels, tens of minutes before and hours after SAH induction. To automate data analysis as much as possible, we developed a complete software, made available on GitHub and readily implemented as a Fiji plugin (see Sec. 4.7), providing RBC velocities from manually drawn ROIs.

This allowed (1) to show a change in transit time distributions, (2) to fit transit time distributions in single animals with competing mathematical models, (3) to conclude that a Cauchy PDF is more appropriate than a Gamma one as it better estimates the longer transit times appearing after SAH, and (4) to show that a Cauchy PDF results, as a Gamma PDF, in a malignant capillary heterogeneity state on ${\mathrm{CMRO}}_{2}^{\max}$ maps.

### 4.2 SAH Mouse Model

When it comes to pathophysiological explorations, the relevance of any experimental model comes into question. Here, we chose a rodent one, because, first, the vascular microarchitecture falls into the same category as that of humans,^6^ and second, the cellular responses to SAH seem similar across mammals, including humans.^44^ Then, the choice of experimental SAH induction can also be a matter of debate. Two approaches are most often reported, intracisternal blood injection as used in this study and mechanical filament perforation of the circle of Willis.^21^ We chose to control the blood volume irrupting into the subarachnoid spaces so that at least the induced intracranial pressure variation, a major determinant of the initial drop in cerebral perfusion,^45^ was reproducible across animals and therefore dismissed filament perforation. It has been shown by others that intracisternal blood injection reproduces a number of pathological features found after aneurysmal SAH in humans: for instance, vasospasm, behavioral and functional deficits, neuroinflammation, and neuronal death.^36^^,^^46^^,^^47^ In conclusion, it is reasonable to investigate the changes induced by experimental SAH in a mouse model to infer the pathophysiological properties of the microvascular network.

### 4.3 Data Acquisition and Processing

In the present experimental design, we made use of an upright widefield epifluorescence microscope. This choice enabled the maximization of the field of view and hence the number of sampled blood vessels. It also provided a high acquisition rate, so the mean and median maximal measurable velocities of 4.76 and 4.28  mm/s, respectively, are above the usually reported ones.^20^^,^^21^^,^^32^^,^^34^^,^^38^^,^^48^ For instance, a recent report of sampling from a high number of microvessels found that the distribution’s peak was between 1 and 3  mm/s, and that more than 85% of vessels had velocities lower than 5  mm/s.^48^ Besides, this approach allowed us to record from a large number of capillaries per animal, which was imperative to fit theoretical PDF at single time points from single mouse data. Indeed, because of inter- and intra-individual variability increasing dispersion, pulling data may result in incorrect velocity distribution estimation.

The first limit of our method is that it underestimates RBC velocities in vessels tilted relative to the imaging plane. Measuring the tilt angle from the Z-stack allows us to correct that effect, but as this angle was very low in our sample, the resulting underestimation was considered negligible. A more important limit is that we recorded from superficial cortical vessels only, with restriction coming from the low imaging depth inherent to the technique. This may be at least partially overcome if using a differential multipoint confocal microscope enabling both high frame rate and deeper imaging,^38^ a more standard two-photon microscope approach to sample from a limited number of deep capillaries,^33^^–^^35^ or the recent advances in optical coherence tomography allowing to compute vascular morphology and flux distribution in a large cortical volume.^48^

Because we acquired large amounts of data, we aimed at automating their processing and therefore developed a new software. Indeed, the existing software (1) needed two-photon line scan images and could not convert full-field images into profile-time images,^49^ (2) required a high signal-to-noise ratio, which was not the case in wide-field images,^50^ or (3) was not accompanied with documentation.^51^

The internal validity of the present software was manually verified by comparing the measured angle to the visible streaks in the profile-time images. Automated verification is also available using two out of four possible metrics: (1) the separability, defined as the maximum variance of the radon transforms divided by the average variance, (2) the full-width half max of the radon transform variances, (3) a correlation coefficient computed as the average correlation between each line of the Sobel filtered profile-time image that is orthogonal to the measured streak angle and the mean of those lines, and (4) the standard deviation evolution computed as the ratio of the standard deviation of the Sobel filtered profile-time image that underwent a 2×2 binning, divided by the same measure without the binning. After partitioning the measured angles with thresholding values of any two out of these four metrics, the Matthews correlation coefficient (MCC) is computed for each pair of thresholding values, and the pair that maximizes the MCC is selected to automatically separate accurate measures from inaccurate ones. External validity of the data analysis pipeline was also confirmed, as obtained RBC velocities were in the same range as published values.^32^^,^^34^^,^^35^^,^^38^

### 4.4 Transit Time Distributions and Implications

#### 4.4.1 RBC velocities

RBC velocities analysis showed results consistent with the SAH literature. In particular, the mean RBC velocity before SAH of 0.95  mm/s was very close to those reported by McConnel and colleagues (0.8  mm/s) and Anzabi and colleagues (0.82  mm/s).^20^^,^^21^ The mean drop of around 50% after SAH was also consistent with these studies, describing a reduction of ≈30% and ≈46%, respectively. The inter-individual variability observed in our animals is not a surprising result, especially given the high inter-subject variability observed in SAH patients.

#### 4.4.2 Capillary transit time distributions

Computed transit times were in the reported order of magnitude.^25^ As expected from the evolution of velocities distributions, transit time distributions changed after SAH. Mean transit time (MTT) and the coefficient of variation “capillary transit time heterogeneity” (CTH) over MTT both showed an increase. This result was clear in two animals and much less pronounced in the third one. This last case was indeed more similar to the constant CTH/MTT ratio reported by Anzabi et al.^21^ In the first situation, differences with the literature may arise from the different time points at which measures have been performed. Indeed, in the cited work, results were obtained 4 days after SAH, as opposed to the tens of minutes following SAH induction in the present work.

In the case of impaired oxygenation resulting from increased CTH/MTT, our results would corroborate a subacute evolution toward DCI.^17^ To explore this hypothesis further,^27^ one needs to first choose a transit times PDF^28^ and implicitly of an underlying microvascular network structure. So far, experimental data were lacking to support the choice of a given PDF.^25^^,^^28^

#### 4.4.3 Transit times PDF

Because we sampled from a high number of capillaries, we were able to fit experimental data with theoretical PDFs. We selected the Gamma PDF, an extensively used PDF in this field of research.^21^^,^^27^^,^^28^ We decided to also evaluate the Cauchy PDF because it emerges from a formal biophysical model.^25^^,^^29^ Gamma PDF appeared to correctly fit data from the pre-SAH period. However, it could not fit both the short and the long transit times composing the full spectrum after SAH. Gamma PDF was first described to fit plasmatic dye transit time in the macrocirculation after a bolus injection.^52^ It has also been reported to be suitable to fit dye transit time in the microcirculation.^53^ In both cases, recirculation of the dye made it difficult to assess long transit times. Studies that have used this PDF have hypothesized it to describe RBC transit times in a capillary network, but so far experimental data were not available, and those from the present work do not support its use, at least in this pathological condition. However, Cauchy PDF correctly captured the two behaviors, i.e., before and after SAH induction.

To estimate PDF sensitivity to capillary lengths, which could be incorrectly estimated in the absence of specific vascular labeling,^4^ we computed transit times using a fixed capillary length, as common in the field,^21^^,^^25^^,^^27^ and using a realistic log-normal distribution.^41^ Distributions were again better fitted with Cauchy than Gamma PDF, confirming that transit time distributions mainly arise from velocities that span more than 2 orders of magnitude, as suggested by Goirand et al.^25^

Other PDFs have also been explored.^21^ Some of them show good statistical agreement with our data and hence are provided as Supplementary Material. However, further theoretical work is needed to assess their biophysical relevance.

Cauchy PDF was described to approximate transit times in synthetic networks built from regular random graphs.^25^^,^^29^ The good match between this PDF and data is of particular interest. Indeed, it tends to validate the biophysical model based on regular random graphs and suggests that other inferences, such as oxygen distribution in various conditions, may be of pathophysiological relevance and deserve further theoretical and experimental explorations.

#### 4.4.4 Maximal ${\mathrm{CMRO}}_{2}$

Transit times PDFs have been used in models to calculate maximal ${\mathrm{CMRO}}_{2}$ and in particular to build ${\mathrm{CMRO}}_{2}$ maps as a function of CTH and MTT.^27^ In such maps, given a Gamma PDF, a “malignant CTH” state is described, in which a decrease in MTT would result in a decrease, and not an increase, in maximal ${\mathrm{CMRO}}_{2}$ due to an elevated CTH.^27^ It has been shown that not any PDF would be associated with such a malignant CTH state.^28^ For this reason, we evaluated the maximal ${\mathrm{CMRO}}_{2}$ for a Cauchy PDF of transit times. Interestingly, a malignant state was also visible, supporting the initial prediction made by Østergaard et al.^17^ that early brain injury may impact RBC flux distribution in the microvascular network and eventually lead to DCI. Further work measuring from deep cortical vessels is needed to infer the maximal ${\mathrm{CMRO}}_{2}$ in these volumes. However, if the theoretical biophysical network underlying Cauchy PDF were to be correct,^25^^,^^29^ PDF would apply to the entirety of the microvascular network, such that inference of a malignant CTH state would stand.

### 4.5 Limits

It has been shown that anesthetic agents modify cerebral hemodynamics,^38^^,^^54^^,^^55^ and therefore, experimental replication in awake animals may be of interest. However, one would raise the concern of the animal’s welfare as SAH induction would doubtlessly constitute a painful procedure. Second, it is unlikely that conclusions drawn from data measured under general anesthesia would not be usable in a more general framework. Indeed, even if the absolute values are likely to be different in awake animals, it is reasonable to expect that the flow distribution is somehow conserved, especially for long transit times as low-flow capillaries are presumably irreversibly damaged in the time frame of microscopic recordings. As such, previous studies also were conducted in anesthetized animals.^20^^,^^21^ Finally, because we conducted experiments in three animals only and did not perform similar measurements without SAH induction, we suggest caution when interpreting the origin of the observed distribution changes, even though results are in good agreement with theoretical^17^^,^^27^ and experimental^21^ work. Other limits were discussed in the previous paragraphs.

### 4.6 Perspectives

Several exciting experiments may continue the present work. First, chronic RBC flux recordings would allow us to characterize the evolution of MTT, CTH/MTT, and transit time distribution. Second, the use of an oxygen biosensor^56^^,^^57^ would allow us to determine the relationship between transit time distribution and tissue oxygenation, thus refining theoretical models. Third, multicolor fluorescence imaging would allow simultaneous blood flow measurement and functional imaging from specific cellular populations. For instance, neuronal depolarization that is supposed to accompany hypoxia^58^^–^^60^ could be studied in light of modifications in capillary flux. Alternatively, the role of pericytes in reducing capillary flux after transient ischemia^4^^,^^7^^,^^61^ could be explored. Such studies would help identify the origin of capillary occlusion, being pericyte constriction,^17^ adhesive leucocytes,^19^ microthrombi,^15^ or any other microvascular component, providing potential therapeutic targets. Finally, preclinical studies could be performed in this animal model as current drugs for preventing or treating DCI are scarce and even controversial.^11^^,^^12^ To this end, new microscopy methods^48^^,^^62^ allowing recording from large volumes may be of great interest and further emphasize the importance of technological development.

### 4.7 Conclusion

The present work proposes that capillary network functional properties are of high interest when studying neurological diseases. It emphasizes the importance of going back and forth between experimental and theoretical efforts, to refine biophysical models and better explain pathophysiological phenomena. It also provides user-friendly software as a Fiji plugin to help analyze RBC velocities from large numbers of vessels in SAH and other pathological models.

## Supplementary Material

10.1117/1.NPh.12.S1.S14612.s01

## Data Availability

The data were analyzed using a toolset and a plugin made for Fiji^63^ (https://fiji.sc). The toolset allowing alignment of images intra- and inter-stacks can be found at https://github.com/ychastagnier/Alignment and is based on the TurboReg^64^ plugin. The plugin allowing measurement of RBC velocity can be found at https://github.com/ychastagnier/Red-Blood-Cell-velocity. Raw images are available upon request.
