# Supplementary material for: Semi-automated analysis of cerebral capillary red blood cell velocities allows modeling of transit time distribution after experimental subarachnoid hemorrhage in mice
Source: Neurophotonics. 2025 May 15;12(Suppl 1):S14612. doi: 10.1117/1.NPh.12.S1.S14612 (PMC12081204; doi:10.1117/1.NPh.12.S1.S14612)
Supplement: Supplementary file 1 [file NPh_012_S14612_SD001.pdf]

Semi-automated analysis of cerebral capillary red blood cells velocities allows modeling of transit times distribution after experimental subarachnoid hemorrhage in mice.

Kévin Chalard[a,b,1], **Yan Chastagnier**[a,c,d,1], **Julie Perroy**[a], **Vivien Szabo**[a,b,*]
[a]IGF, Univ. Montpellier, CNRS, INSERM, Montpellier, France
[b]Department of critical care and anesthesiology Gui de Chauliac, CHU Montpellier, Montpellier, France
[c]LIRMM, Univ. Montpellier, CNRS, Montpellier, France
[d]L2C, Univ. Montpellier, CNRS, Montpellier, France
[1]these authors contributed equally to this work

Supplemental material.

1 Uncertainties measurement

1.1 Resolution uncertainties.

Resolution is limited either by the size of the pixel or the diffraction limit. In our case, pixel size was 0.57 µm, while the diffraction limit d is given by the equation:

$$d = \frac{\lambda}{2\text{NA}} = \frac{0.6\mu m}{2 * 0.3} = 1\mu m$$

The limiting factor was therefore the diffraction limit $d = 1$ µm. We obtained the uncertainty by dividing d by the length of the ROIs. Distribution of these errors is given in Figure S1 B-C. Mean uncertainty was 1.34%, median 1.22%.

1.2 Velocity underestimation in tilted vessels.

Because we were acquiring T-stacks using a widefield modality and drawing our ROIs by maximizing their length, some vessels could be not perfectly horizontal, which would impair the measure of velocity by underestimating it. To assess the extent of this underestimation, we measured the angle between the vessels and the imaging plane on a Z-stack.

To do so, each ROI was first manually registered to place it correctly on X and Y axis. Then, an image was generated where each horizontal line was the ROI profile for a given z-position, using the dynamic reslice function of Fiji. For each column, the pixel values were extracted in a vector. The vector was filtered with a moving

average of length 11, then the position of the maximum was computed. Another vector was filled with the maximum position of each column. The vector was filtered consecutively with a moving median of length 15 then a moving average of length 11. The vector was then fitted with a straight line.

A manual verification was then performed to validate the straight lines or redraw them. For that, both the filtered vector and the straight line were traced on the z-profile image. If the line was correct, the angle to the horizontal was directly measured. If the line was not following the vessel, for example when a big vessel was crossing the ROI path, generating a signal much bigger than the capillary and deviating the line, it was redrawn and the angle to horizontal was updated with this new value.

Distribution of the angles measured with this method is shown in Figure S1 D. The resulting underestimation in ROIs length and therefore velocities is given as probability distribution and cumulative distribution in Figure S1 E-F. Mean length increase factor was 1.56% , median 0.6%.

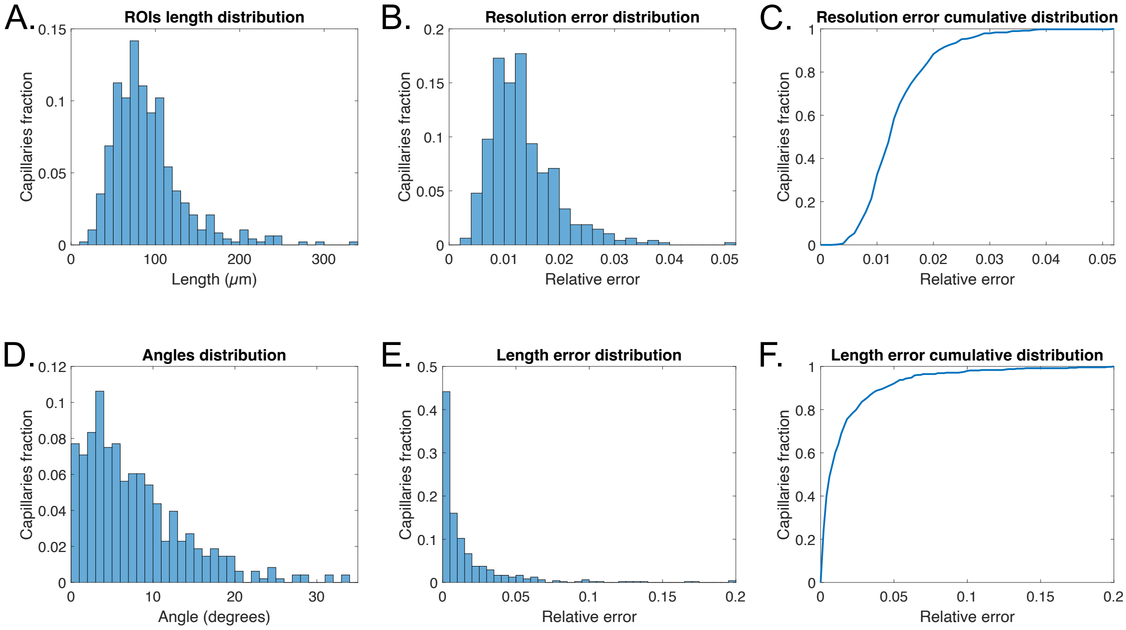

Fig S1 Uncertainties in RBC velocity calculation. **A.** Drawn ROIs length distribution. **B.** Resolution error distribution. **C.** Resolution error cumulative distribution. **D.** Capillaries angle distribution relative to horizontal. **E.** Resulting distribution of ROIs length underestimation error due to not in plane capillaries. The error is the same for the velocities. **F.** Cumulative distribution of ROIs length underestimation error.

2 Velocities

It was possible to perform longitudinal RBC velocity measurements in single capillaries. As an example, and for readability, a subset of 15 capillaries is presented in Figure S2.

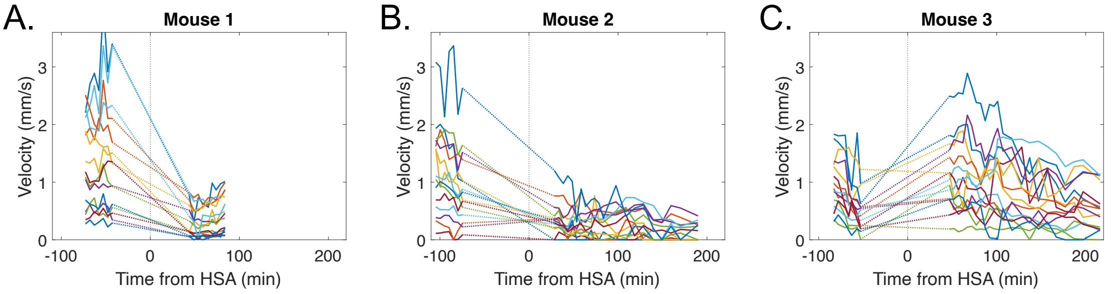

Fig S2 Longitudinal measure of RBC velocities. A.B.C. RBC velocities in single capillaries against time, before and after subarachnoid hemorrhage in each animal. To optimize readability, a subset of 15 capillaries from each mouse is presented. Dotted part corresponds to the surgical procedure, no data points were acquired during this period.

We present fits of velocities distributions in Figure S3, using inverse Gamma[1] and Cauchy[2] PDF.

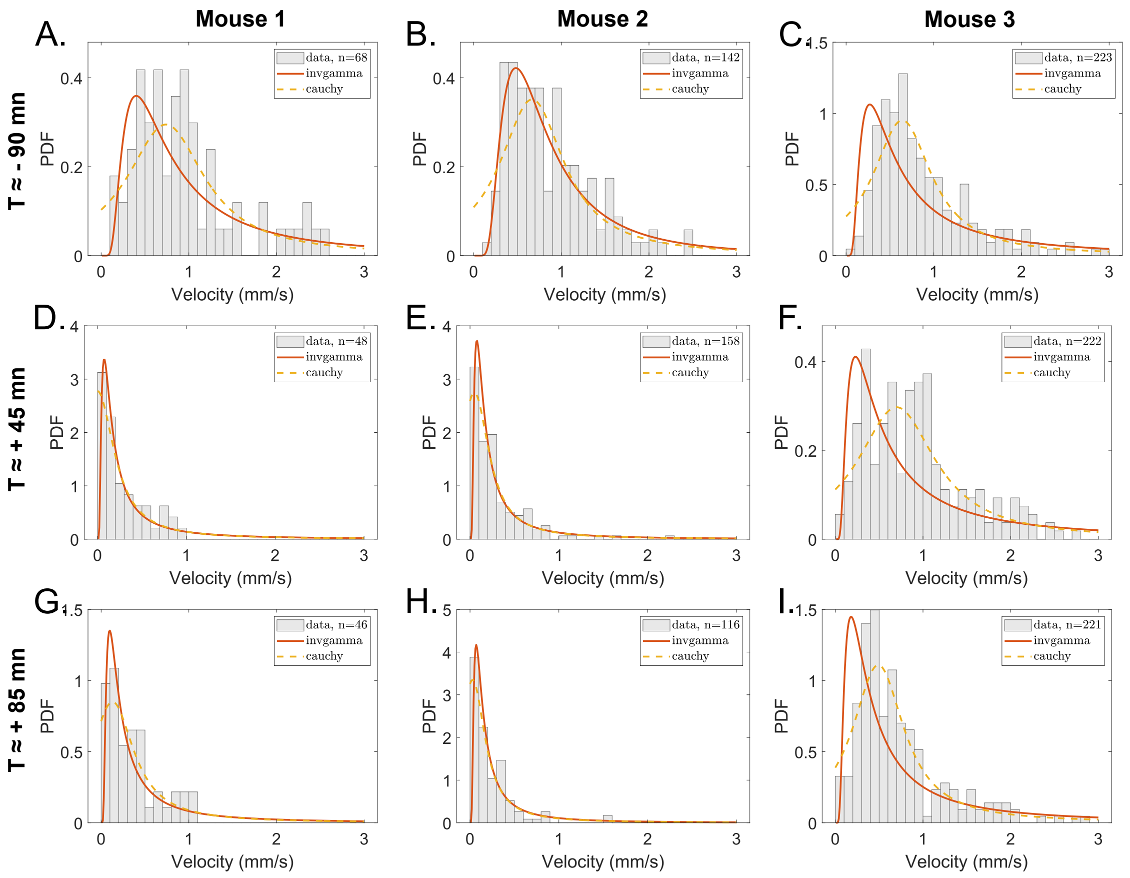

Fig S3 RBC velocities distribution with fits for inverse Gamma and Cauchy PDFs. A.B.C. First timepoint for each mouse at baseline, before subarachnoid hemorrhage (SAH) induction. **D.E.F.** Second timepoint selected at around 45 minutes after SAH induction. **G.H.I.** Third timepoint selected at around 85 minutes after SAH induction.

3 Transit times distributions

It has been suggested that transit times distribution strongly depends on velocities, spanning more than two orders of magnitude, and moderately on capillary length.[2] In order to evaluate this, we computed transit times using 3 estimations of capillary lengths, 1) the length of the ROI (see main text), 2) a constant length of 50 µm (Figure S4) as in the work from Goirand and colleagues,[2] and random lengths from a realistic log-normal distribution with a median around 50 µm (one example is shown in Figure S5) as measured by Blinder and colleagues.[3]

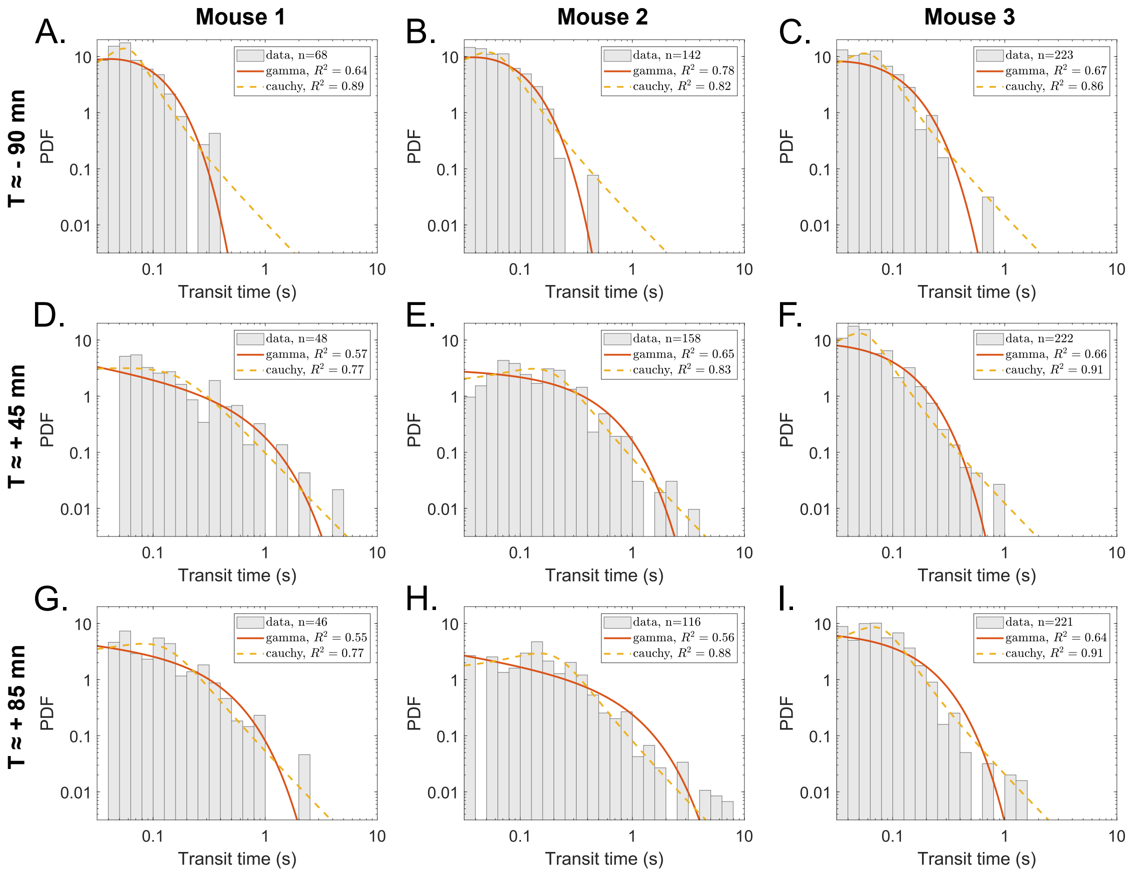

Fig S4 RBC transit times distribution computed with constant capillary length of 50 µm, fitted with Gamma and Cauchy PDFs. A.B.C. First timepoint for each mouse at baseline, before SAH induction. **D.E.F.** Second timepoint selected at around 45 minutes after SAH induction. **G.H.I.** Third timepoint selected at around 85 minutes after SAH induction.

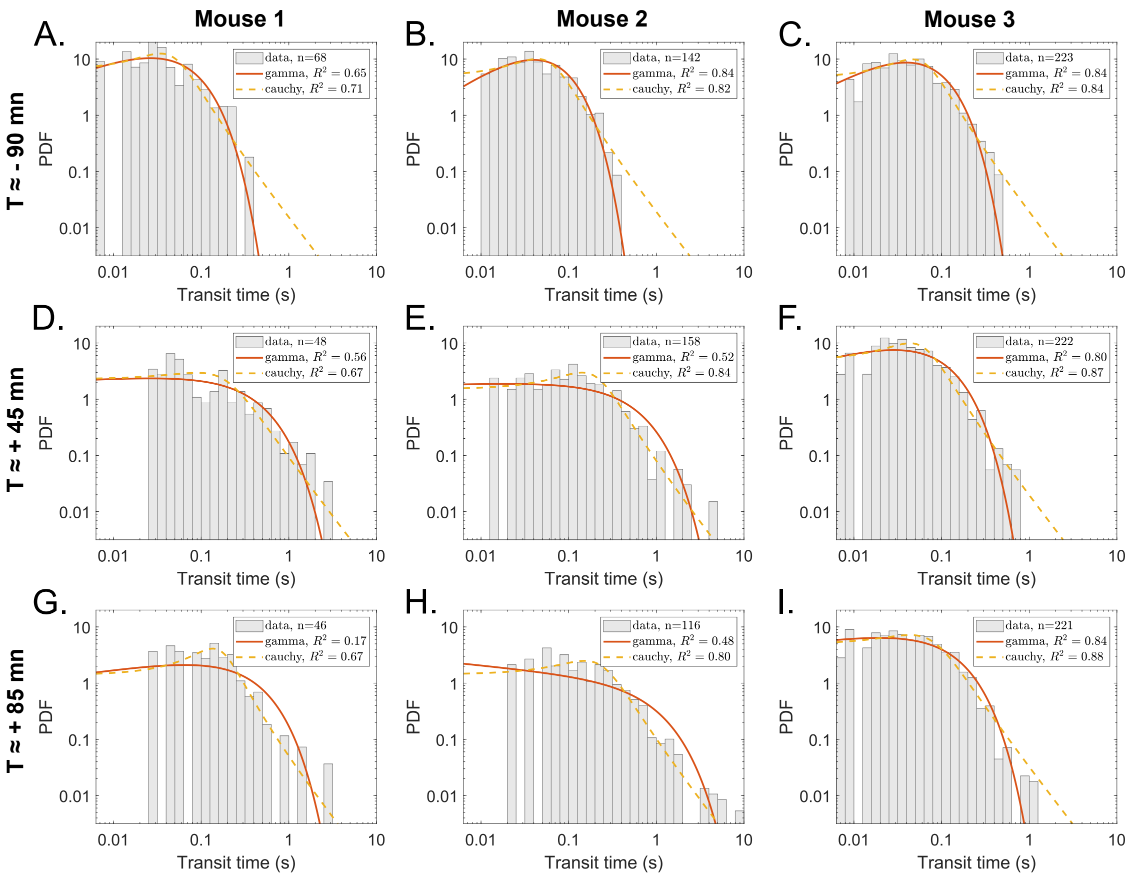

Fig S5 RBC transit times distribution computed with random capillary length distributed log-normally around 50 µm, fitted with Gamma and Cauchy PDFs. A.B.C. First timepoint for each mouse at baseline, before SAH induction. **D.E.F.** Second timepoint selected at around 45 minutes after SAH induction. **G.H.I.** Third timepoint selected at around 85 minutes after SAH induction.

Besides PDFs emerging from biophysical models of underlying vascular networks, i.e. Gamma[4] and Cauchy PDFs,[2] other functions may fit transit times distributions.[1] We evaluated the goodness of fit of theses PDFs and present the results in Figure S6 for potential future use. However, as of today, they do not provide further information on the vascular network's properties.

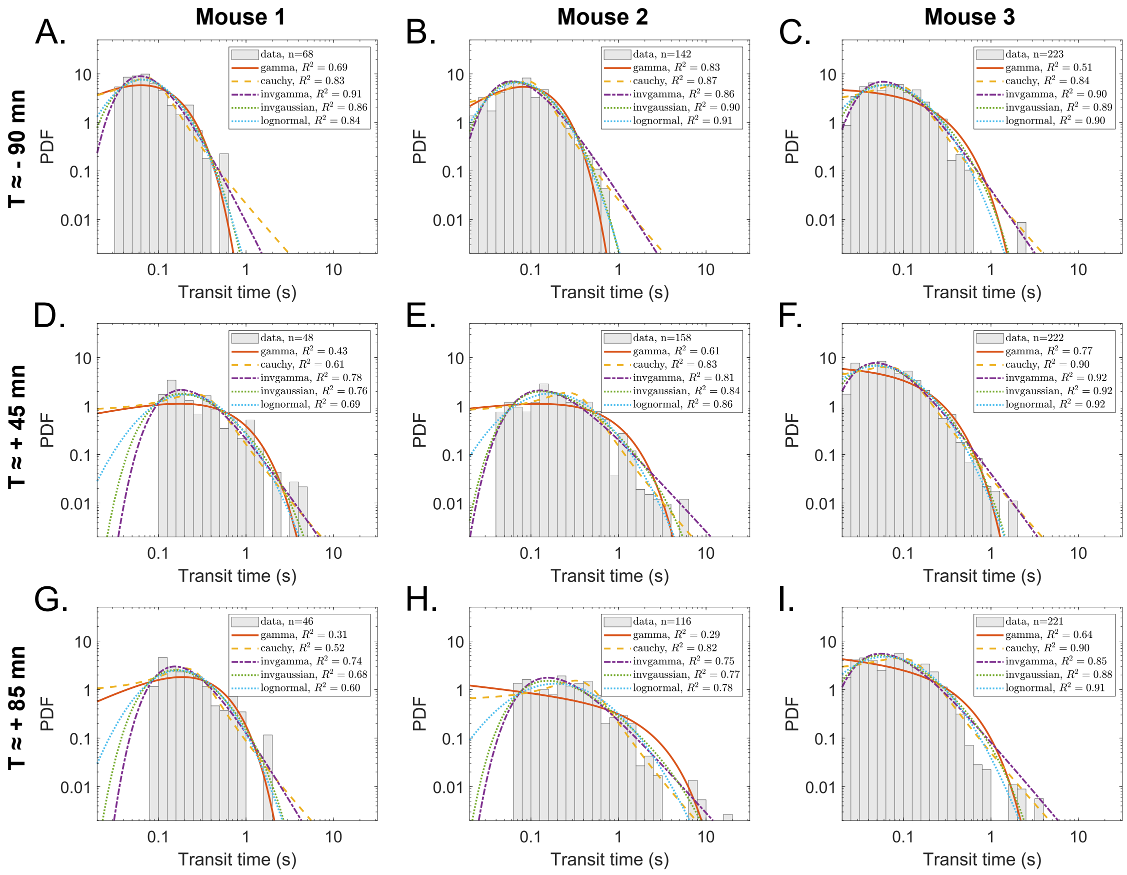

Fig S6 RBC transit times distribution computed with ROI length, fitted with Gamma, Cauchy, Inverse Gamma, Inverse Gaussian and log-normal PDFs. A.B.C. First timepoint for each mouse at baseline, before SAH induction. **D.E.F.** Second timepoint selected at around 45 minutes after SAH induction. **G.H.I.** Third timepoint selected at around 85 minutes after SAH induction.

References

1 H. Angleys, L. Østergaard, and S. N. Jespersen, "The effects of capillary transit time heterogeneity (CTH) on brain oxygenation," Journal of Cerebral Blood Flow & Metabolism **35**, 806–817 (2015).

2 F. Goirand, T. Le Borgne, and S. Lorthois, "Network-driven anomalous transport is a fundamental component of brain microvascular dysfunction," Nature Communications **12**, 7295 (2021).

3 P. Blinder, P. S. Tsai, J. P. Kaufhold, et al., "The cortical angiome: an interconnected vascular network with noncolumnar patterns of blood flow," Nature Neuroscience **16**, 889–897 (2013).

4 H. K. Thompson, C. F. Starmer, R. E. Whalen, et al., "INDICATOR TRANSIT TIME CONSIDERED AS A GAMMA VARIATE," Circulation Research **14**, 502–515 (1964).
